# Cetylpyridinium chloride inhibits hepatocellular carcinoma growth and metastasis through regulating epithelial-mesenchymal transition and apoptosis

**DOI:** 10.1371/journal.pone.0310391

**Published:** 2024-09-20

**Authors:** Kundi Cai, Yihui Fang, Yanan Zhang, Jie Liu, Qinong Ye, Lihua Ding, Xianfeng Cai

**Affiliations:** 1 Jiangxi Normal University, Jiangxi, China; 2 Laboratory of advanced biotechnology, Department of Cell Engineering, Beijing Institute of Biotechnology, Beijing, China; National University Singapore Yong Loo Lin School of Medicine, SINGAPORE

## Abstract

Hepatocellular carcinoma (HCC) is characterized by a lack of obvious clinical features in the early stages and is likely to progress to advanced HCC. Advanced HCC is a highly malignant tumor. However, there are few treatment options for advanced HCC. Therefore, screening for new drugs that target HCC will provide a new approach to the treatment of HCC. The CCK8 assay was performed to screen compounds inhibiting HCC cell proliferation and to evaluate the IC_50_ (half-maximal inhibitory concentration) of compounds on cell lines. Colony formation assay was used to determine HCC cell proliferation. The effect of compounds on HCC cell migration and invasion were analyzed using wound healing and transwell assays, respectively. Tumor growth and metastasis were assessed *in vivo* in a xenograft mouse model. Flow cytometry was carried out to measure apoptotic cells. Reverse transcription and quantitative real-time polymerase chain reaction (RT‒qPCR) and Western blot were performed to examine the expression of epithelial-mesenchymal transition (EMT)- and apoptosis-related genes. Through large-scale screening, we have discovered the anti-tumor activity of cetylpyridinium chloride (CPC) against HCC cells. CPC inhibited the proliferation, invasion and metastasis of HCC cells. Cancer cells are more sensitive to CPC than normal cells. CPC suppressed HCC tumor growth and metastasis *in vivo*. Mechanistically, CPC promoted apoptosis of HCC cells by affecting the expression of apoptosis-related genes, and inhibited HCC invasion and metastasis by suppressing EMT and expression of EMT markers. Our investigation showed that CPC significantly inhibited HCC cell proliferation, invasion and metastasis *in vivo* and *in vitro*, by inducing the expression of apoptosis-related genes and inhibiting expression of EMT markers, suggesting that CPC is a potential agent for HCC treatment.

## Introduction

Liver cancer is the sixth most common cancer and the third leading cause of death worldwide. Hepatocellular carcinoma (HCC) is the most common form of primary liver cancer. In 2019, there will be approximately 747,000 HCC cases worldwide, with a median survival of 6–10 months [1–5]. Viruses (hepatitis B virus and hepatitis C virus), alcohol, metabolic abnormalities (fatty liver) and dietary toxins (aflatoxin, aristolochic acid, etc.) are risk factors for HCC [6, 7]. In recent years, the incidence of HCC caused by metabolic abnormalities, non-alcoholic fatty liver disease and non-alcoholic steatohepatitis has increased.

Most cases of HCC are diagnosed at an advanced stage and the chance of surgical remission has been lost [8, 9]. Unresectable HCC is usually treated with trans-arterial chemoembolization (TACE) [10]. However, the success rate of TACE is only 40% [10, 11]. Chemotherapy is therefore one of the main treatments for liver cancer [8]. Sorafenib, the first drug approved by the Food and Drug Administration for use in patients with unresectable HCC, often develops drug resistance [12–14]. Doxorubicin (DOX) is the first-line chemotherapy for HCC, which inhibits HCC tumors by inhibiting DNA replication and translation, but DOX has a survival benefit of only 3.0 to 4.1 months [15, 16]. Platinum-based chemotherapeutics (such as cisplatin, carboplatin and oxaliplatin) provide only a marginal survival benefit in the treatment of HCC due to drug resistance [17]. The fluoropyrimidine 5-fluorouracil (5-FU) inhibits DNA and RNA synthesis by mis-incorporating fluoronucleotides, but has a low response rate and no effect on overall survival for HCC patients [18]. Chemotherapeutic drugs such as DOX are not only ineffective, but also cause toxic side effects and drug resistance within a year of completion treatment [16]. The combination of regorafenib, ramucirumab and cabozantinib has shown partial activity in patients who have failed sorafenib [19–24]. However, due to the diversity and heterogeneity of HCC, no effective drug has been found to prolong the lives of HCC patients. Therefore, new drugs treating liver cancer need to be developed.

We screened 2067 drugs and found that cetylpyridinium chloride (CPC) inhibited the proliferation, migration and invasion of HCC cells. Cetylpyridinium chloride is synthesized by esterifying cetyl alcohol with pyridine hydrochloride, followed by purification through crystallization to obtain the final product. Cetylpyridinium chloride enters cells primarily by passive diffusion, driven by its amphiphilic structure and interactions with cell membranes. CPC is a quaternary ammonium surfactant with broad-spectrum antimicrobial activity [25]. Studies have shown that CPC inhibits the survival of human breast tumor cells and kills glioblastoma cells in a dose-dependent manner [26, 27]. The mechanism by which CPC inhibits tumor cell activity includes activation of AMP-activated protein kinase (AMPK) and activation of caspase-3, thereby promoting apoptosis [26]. These results suggest that CPC has potential for cancer treatment, but the function and mechanism of CPC inhibition of HCC cells have not been reported.

In this study, we investigated and found that CPC inhibited HCC tumor growth and metastasis *in vitro* and *in vivo* by inducing apoptosis-related gene expression and inhibiting expression of EMT markers, suggesting that CPC may be a potential drug candidate for the treatment of liver cancer.

## Materials and methods

### Cell culture

All cell lines used in this study were obtained from the American Type Culture Collection (ATCC, USA). MCF-7, ZR-751, T47D, BT474, MDA-MB-231, MDA-MB-468, MDA-MB-436, A549, H1299, HepG2, MHCC-97H, SMMC7721, 293T, LO2, HMEC, and NIH3T3 were cultured in DMEM (Dulbecco’s modified Eagle’s medium, Invitrogen, Carlsbad, CA, USA) supplemented with 10% fetal bovine serum (Gibco, Carlsbad, CA, USA) and 1% penicillin-streptomycin solution 100X (Corning, CA, USA) in a humidified atmosphere with 5% CO2 at 37°C. L4000 was purchased from TargetMol Chemicals Inc. Compounds were dissolved and diluted in normal saline for cell culture studies. Anti-Bax (#AF0057), anti-p53 (#AF0255) and anti-PARP (#AF1657) were purchased from Beyotime Biotechnology. Anti-cleaved caspase-3 (#AF7022) was purchased from Affinity Biosciences. Anti-β-actin antibody (#sc-47778HRP) was purchased from Santa Cruz Biotechnology.

### Compound screening

We screened 2067 compounds using compound libraries (L4000, TargetMol). A total of 3000 HepG2 cells were plated in 96-well plates and treated with 10 μM compounds. After 48 h incubation, the CCK8 assay was performed according to the manufacturer’s protocols (Dojindo Laboratories).

### IC_50_ assays

The IC_50_ of CPC on various cell lines was evaluated using the CCK8 assay. Cell lines in complete cell culture medium were seeded in 96-well plates (100 μL per well) at 3000 cells/well, allowed to attach overnight and then treated with CPC. We evaluated the IC_50_ values of CPC at six different concentrations. The compounds were prepared in complete cell culture medium and 100 μL of CPC was added to each well for 48 h. Subsequently, 100 μL of cell culture medium containing 10% CCK-8 solution was added to the cultured cells and the mixture was incubated at 37°C and 5% CO_2_ for 1 h. OD values were measured at 450 nm using a microplate reader. The mean of three independent experiments was used to assess the validity of the results. The IC_50_ value was evaluated using GraphPad Prism 7.

### Cell proliferation and colony formation assays

Approximately 3000 cells per well were seeded in 96-well plates and grown at 37°C for the indicated times and treated with CPC (0 μM, 1 μM, 2 μM, 5 μM). Cell numbers were determined using a CCK-8 kit (Dojindo Laboratories) according to the manufacturer’s protocols. The absorbance at 450 nm of each well was measured using a microplate reader. Absorbance was measured every 24 hours for 4 days.

The colony formation assay was performed using MHCC97-H and HepG2 cells treated with CPC. Cells treated with CPC for 48 h were plated in triplicate in six-well dishes at 2000 cells per well and allowed to grow for 10–14 days. The number of colonies greater than 1.0 mm in diameter was scored.

### Cell migration and invasion

For the cell migration assay, cells were plated at 90% density in 6-well plates. Confluent monolayers of cells were scraped mechanically with a 200 μL pipette tip. The debris was washed three times with PBS and incubated with DMEM without FBS supplemented with CPC (0 μM, 1 μM, 2 μM, 5 μM). Cell migration rates were further investigated by calculating wound widths at 0 h and 24 h.

For the cell invasion assay, Matrigel (BD Biosciences) was melted on ice and then dropwise to the upper surface of the Transwell chamber (Corning). Cancer cells were washed three times with PBS and added to medium supplemented with CPC (0 μM, 1 μM, 2 μM, 5 μM) at 10,000 cells per well. After 24 h, cells invading the matrix gel membrane were fixed with 4% paraformaldehyde and then stained with crystal violet. The number of invading cells was counted after photography.

### Cell apoptosis

Cancer cells (5 × 10^3^ cells) were cultured in six-well dishes to adhere overnight and were then treated with CPC for 48 h. The cells were labeled with propidium iodide and Annexin V according to the manufacturer’s instructions (KeyGen Biotech). Apoptotic cells were analyzed by flow cytometry.

### Reverse transcription and Quantitative Real-Time Polymerase Chain Reaction (RT‒qPCR)

Hepatocellular carcinoma cells were cultured in six-well dishes to adhere overnight and then treated with the compound for 48 h. Total RNA was extracted using TRIzol reagent (Invitrogen, Carlsbad, CA) according to the manufacturer’s protocol. Subsequently, 2 mg of total RNA was reverse transcribed into cDNA according to the manufacturer’s recommendations (Takara). mRNAs were determined using a QuantiFast SYBR Green PCR kit on a CFX96 real-time PCR detection system. The relative fold expression of the target, normalized to the corresponding control, was calculated using the comparative Ct method. Primer sequences for RT-PCR were as follows: β-actin, forward:5’-TCGTGCGTGACATTAAGGAG-3’, reverse: 5’-ATGCCAGGGTACATGGTGGT-3’; E-cadherin, forward: 5’-TGCCCAGAAAATGAAAAAGG-3’, reverse: 5’-GTGTATGTGGCAATGCGTTC-3’; N-cadherin, forward: 5’-ATTGGACCATCACTCGGCTTA-3’, reverse: 5’-CACACTGGCAAACCTTCAGG-3’; Slug, forward: 5’- TGTGTGGACTACCGCTGCTC-3’; reverse 5’- GAGAGGCCATTCGGTAGCTG-3’; Vimentin, forward: 5’-TACAGGAAGCTGCTGGAAGG-3’, reverse 5’-ACCAGAGGGAGTGAATCCAG-3’.

### Western blot analysis

Cells were lysed in RIPA lysis buffer containing protease inhibitors for 30 minutes. Equal amounts of protein were separated by 10% SDS-PAGE and transferred to a nitrocellulose membrane. The membrane was blocked for 1 hour, incubated with the indicated antibodies and detected by enhanced chemiluminescence (Promega). The membranes were probed with primary antibodies and then incubated with horseradish peroxidase-conjugated secondary antibodies. Immune complexes were detected using an Immobilon™ Western chemiluminescence HRP substrate (Millipore) and photographed using a Tanon 5200 imaging system.

### *In vivo* analysis of tumor growth and lung metastasis

Animal experiments were approved by the Institutional Animal Care Committee of the Beijing Institute of Biotechnology, and we have obtained informed written consent. For in vivo analysis of tumor growth, 5 × 10^6^ HepG2 cells were injected subcutaneously into six-week-old male BALB/c nude mice (Sibeifu, Beijing, China). Mice were randomly divided into groups when tumors reached a volume of ∼50 mm^3^ and injected intraperitoneally with CPC (0, 5, 15, 30 mg/kg) or saline. The diameters of the tumors were measured with calipers at the indicated times. The tumor volume was calculated using the formula: (longest diameter × shortest diameter^2^)/2. Mice were euthanized at the indicated times. To reduce mouse suffering, mice were euthanized with carbon dioxide before the tumor grew to 2 cm in diameter. For lung metastasis analysis, 1 × 10^6^ MHCC-97H-luc cells were injected into the lateral tail vein of six-week-old male BALB/c nude mice. Seven days after injection, mice were randomized into groups and injected intraperitoneally with CPC (0, 5, 15, 30 mg/kg) or saline. After another 30 days, the mice were imaged using an IVIS200 imaging system (Xenogen Corporation, USA). At the end of the experiment, the mice were euthanized with carbon dioxide, tumor tissues were harvested, and the carcasses were weighed and uniformly delivered to the animal facility for proper disposal without risk to the environment or personnel.

### Statistical analysis

Statistical analysis was performed using SPSS 25.0 and GraphPad Prism 7 software. ImageJ was used to calculate mobility. Student’s t-test was used to compare two groups. Data are presented as mean ± standard deviation, and *P* values < 0.05 were considered statistically significant. All *in vitro* experiments were performed in triplicate and repeated 3 times.

## Results

### High-throughput compound screening identifies small molecule inhibitors of HCC cell proliferation

**Fig 1:**
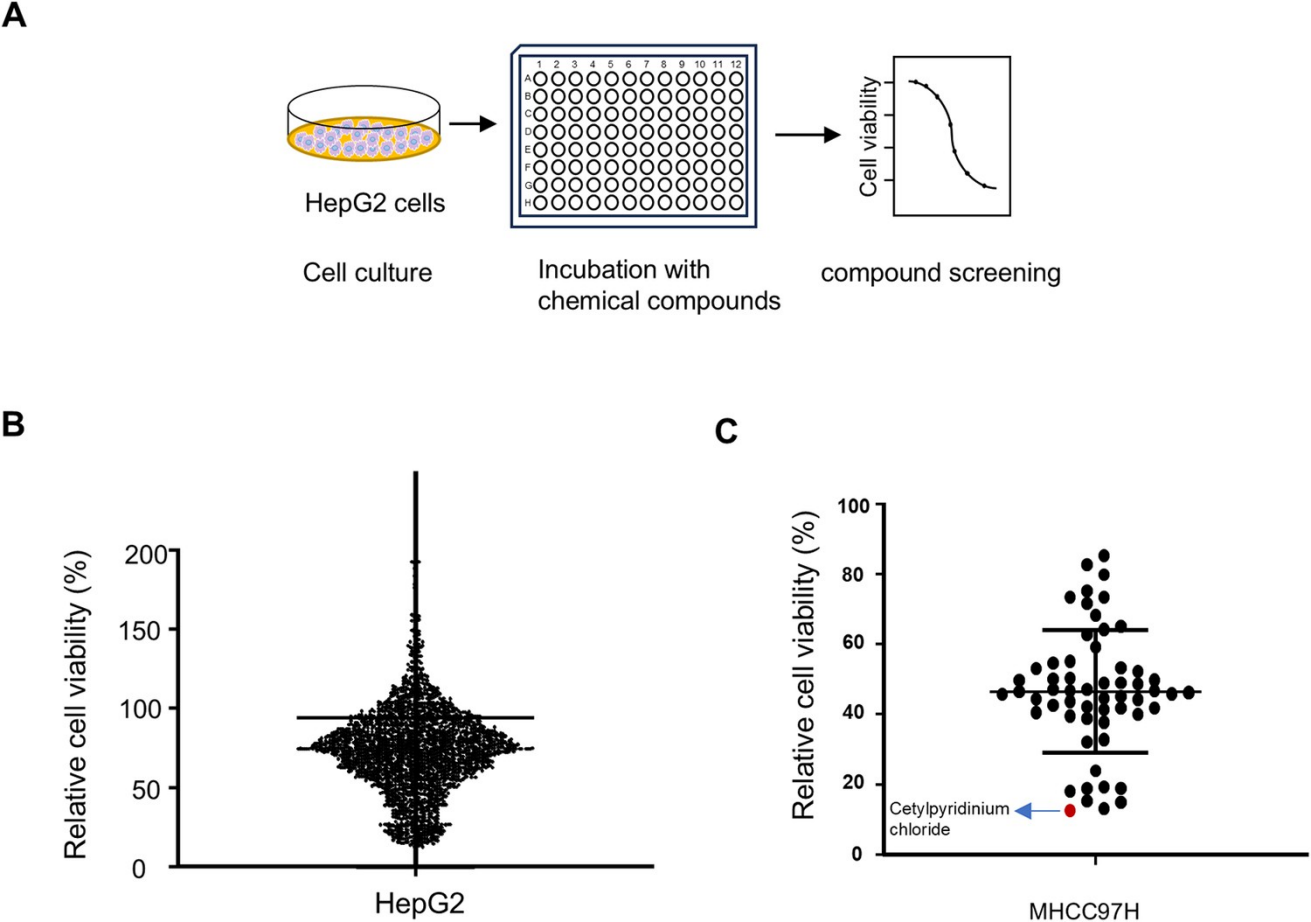
High-throughput screening identifies small molecule compounds for inhibiting HCC cell proliferation. **A** Schematic representation of high-throughput screening of potential drug candidates for inhibiting HCC cell proliferation. **B** Dot-plot of CCK8 screening for compounds that inhibit HepG2 cell proliferation using the L4000 small molecule compound library. Each point is the mean value of the three replicates. **C** Dot plot of CCK8 screening for compounds that inhibit MHCC97H cell proliferation using the 58 small molecule compounds screened from (B). Each dot is the mean of the three replicates.

To identify potential drugs against HCC, we screened small molecule inhibitors of HCC cell proliferation using a compound library (L4000) containing 2067 compounds. In the first round of screening, we examined the effects of the 2067 compounds on HepG2 cell proliferation (Fig 1A and 1B). We found that 58 compounds significantly inhibited HepG2 cell proliferation (>80%), including compounds that have been reported to inhibit HCC cell proliferation, such as pioglitazone, mifepristone, dobutamine, and metformin [28–31]. We further determined the effect of the 58 compounds on the proliferation of the metastatic HCC cell line MHCC97H, and found CPC significantly inhibited the proliferation of MHCC-97H cells (Fig 1C). Since the inhibitory function of CPC in HCC has not been reported, we chose CPC for further study.

### CPC inhibits hepatoma carcinoma cell proliferation

**Fig 2:**
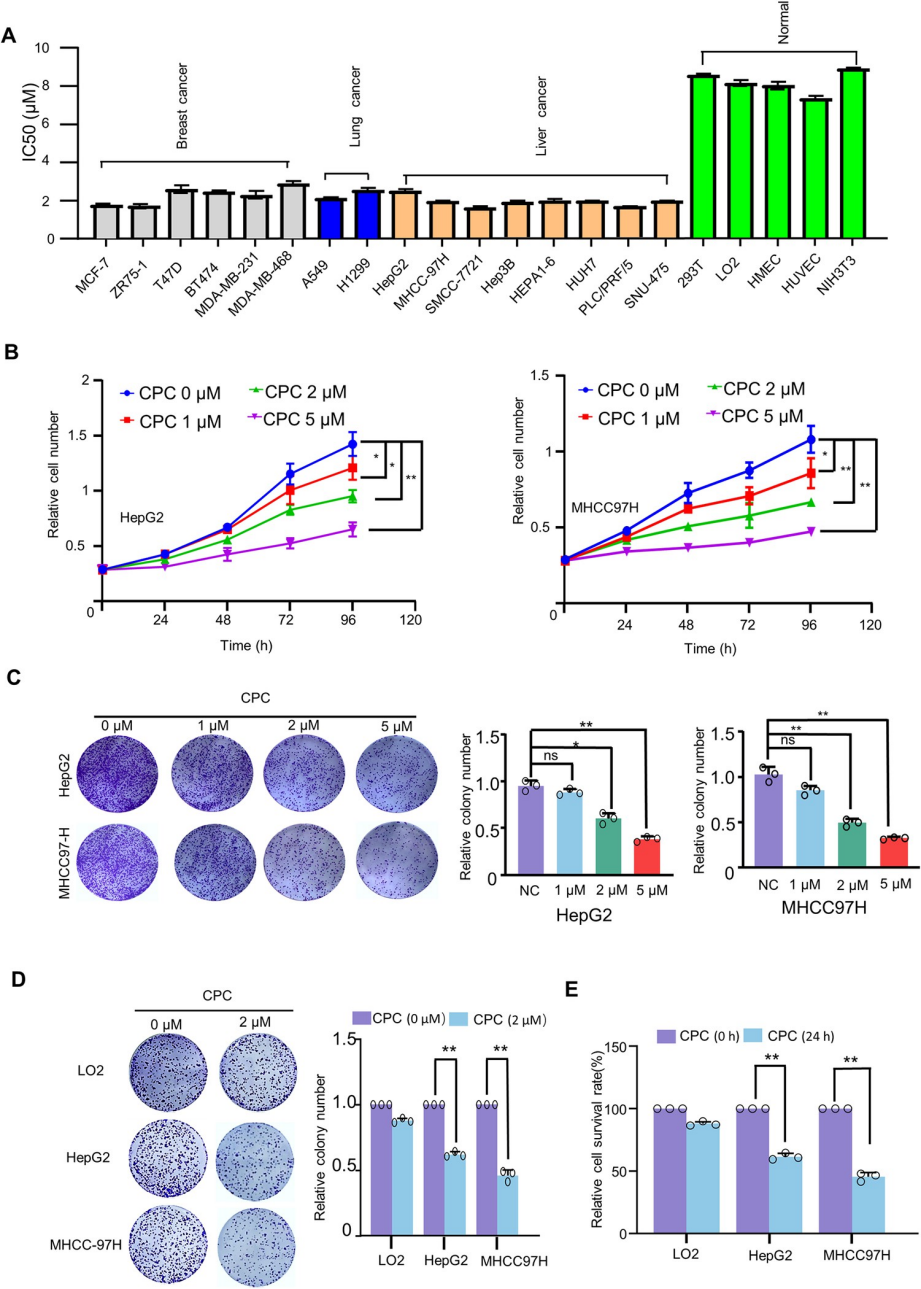
CPC inhibits the proliferation of HCC cells. **A** The IC_50_ values of CPC in 16 cell lines were determined using the CCK8 assay. **B** The proliferation curve of HepG2 and MHCC97H cells treated with different concentrations of CPC or control (normal saline) with indicated times. **C** Colony formation assays for HepG2 and MHCC97H cells treated with different concentrations of CPC or control (normal saline) and stained with crystal violet. Histograms show the number of colonies. Data shown are mean ± SD of triplicate measurements that have been repeated three times with similar results. Student’s t-test was used to compare two groups. **D** Colony formation assays for LO2, HepG2 and MHCC97H cells treated with CPC or control (normal saline) and stained with crystal violet. Histograms represent the number of colonies. Data shown are mean ± SD of triplicate measurements that have been repeated three times with similar results. Student’s t-test was used to compare two groups. **E** CCK8 assay for LO2, HepG2 and MHCC97H cells treated with CPC or control (normal saline). Histograms show the relative number of cells. Data shown are mean ± SD of triplicate measurements that have been repeated three times with similar results. Student’s t-test was used to compare two groups. *p < 0.05, **p < 0.01 versus HepG2 and MHCC97H cells treated with control. ns, no significance.

We used 16 cancer cell lines and 5 normal cell lines to investigate the effect of CPC on cell viability (Fig 2A). We found IC_50_ values in various cancer cell lines ranging from 1.84 to 2.91 μM, indicating that CPC can reduce cancer cell viability without cell type specificity, while IC_50_ values in normal cell lines ranged from 7.12 to 8.83 μM, indicating that cancer cells are more sensitive to CPC than normal cells. In particular, in liver cells, the IC50 in 8 HCC cell lines is significantly lower than that in the normal liver cell line LO2.

Using a cell proliferation assay, we found that CPC inhibited the proliferation of HCC cells in a dose- and time-dependent manner (Fig 2B). Colony formation assays showed that CPC treatment significantly inhibited the colony-formation ability of MHCC97H and HepG2 cell lines compared to the control group (Fig 2C). In addition, we further tested the effect of CPC on normal liver cells LO2 using CCK8 assay and colony formation assay. Compared with HCC cells, CPC had less inhibitory effect on the growth of normal liver cells (LO2) (Fig 2D and 2E). These results suggest that CPC has potent anti-proliferative activity in HCC cells.

### CPC inhibits migration and invasion of hepatocellular carcinoma cells

**Fig 3:**
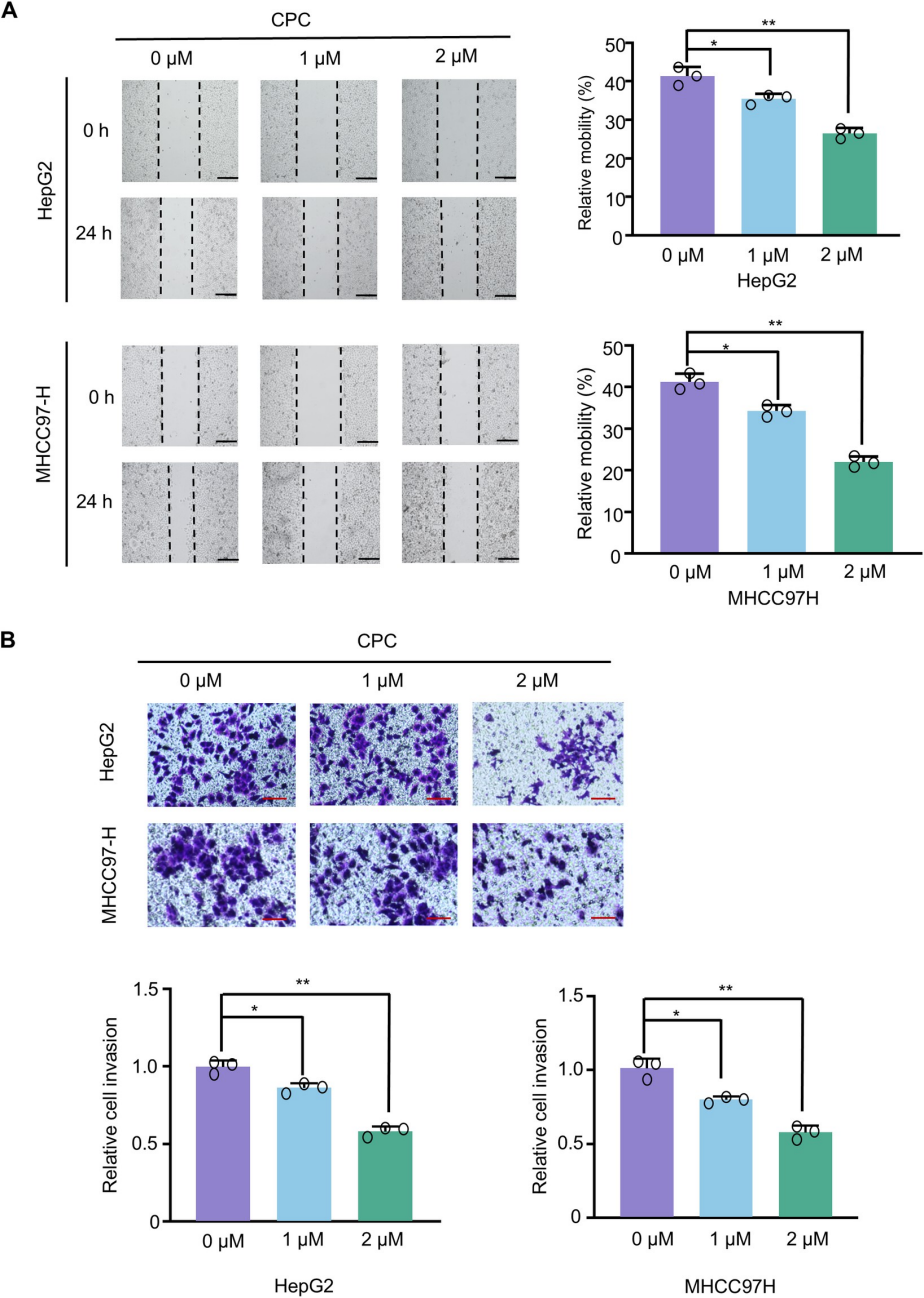
CPC inhibits migration and invasion of HCC cells. **A** Wound-healing assay of HepG2 and MHCC97-H cells treated with different concentrations of CPC or control (normal saline) for 24 h. Histograms show relative cell migration (right panel). Scale bar: 100 μm. **B** Transwell assay of HepG2 and MHCC97H cells treated as in A. Invasive cells were fixed and stained with crystal violet. Histograms show relative cell invasion (bottom). Data shown are mean ± SD of triplicate measurements that have been repeated three times with similar results. Student’s t-test was used to compare two groups. *p < 0.05, **p < 0.01 versus HepG2 or MHCC97H cells treated with control.

We then investigated the effect of CPC on the migration and invasive capacity of HCC cells. Wound-healing assays showed that CPC significantly inhibited the migration ability of HepG2 and MHCC97H cells in a dose-dependent manner (Fig 3A). Transwell assays showed that CPC inhibited HCC cell invasion in a dose-dependent manner (Fig 3B). Therefore, these results indicated that CPC inhibited the migration and invasion of hepatoma carcinoma cells.

### CPC promotes apoptosis in hepatoma cells through regulating apoptosis-related gene expression

**Fig 4:**
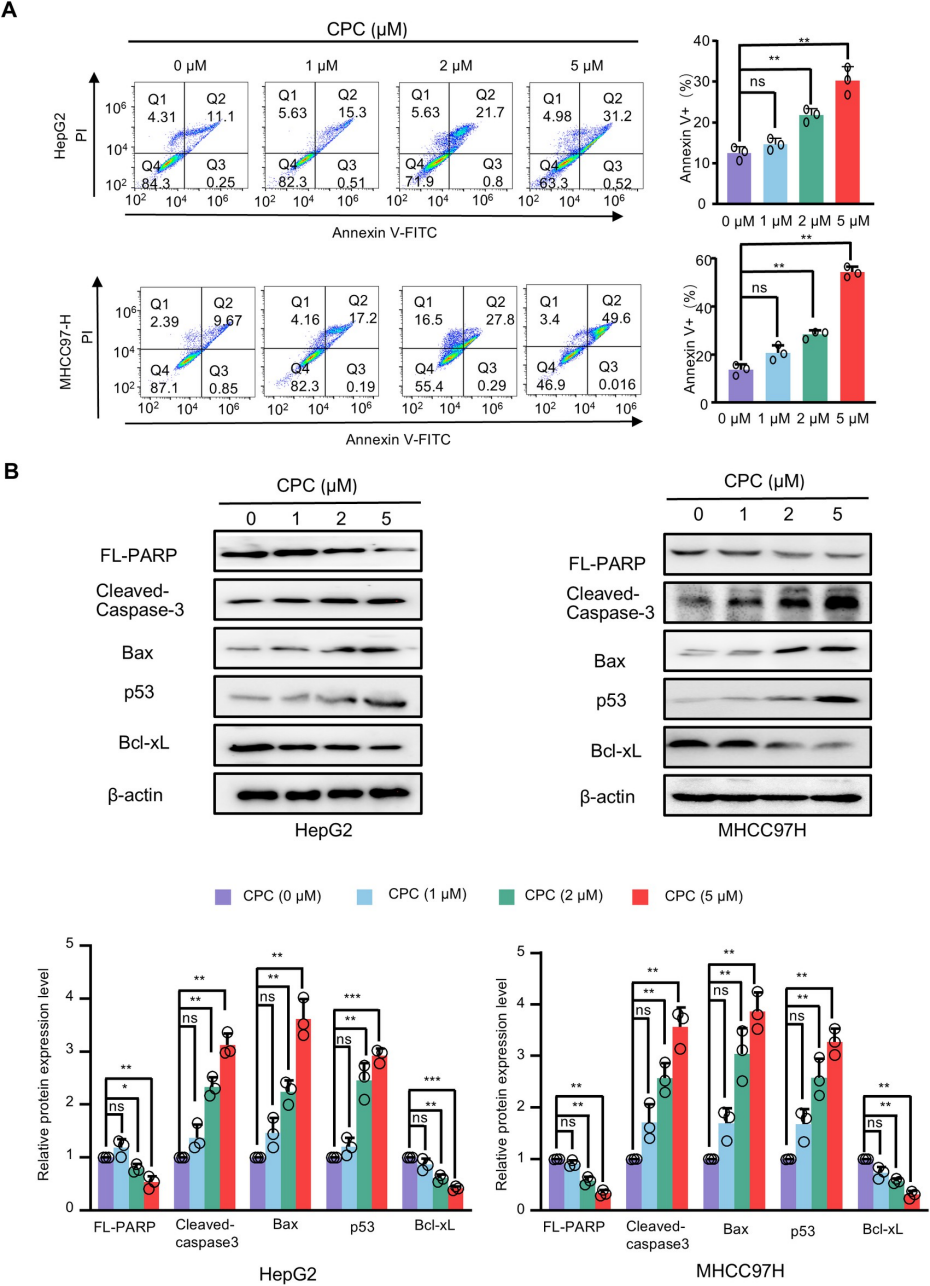
CPC promotes apoptosis of HCC cells and regulates the mRNA and protein expression of apoptosis-related genes. **A** Representative images of flow cytometry analysis of apoptosis in HepG2 and MHCC97H cells treated with different concentrations of CPC or control (normal saline) for 48 h. The graphs show the relative cell apoptosis (early apoptosis and late apoptosis) rates (right panel). Student’s t-test was used to compare two groups. *p < 0.05, **p < 0.01 versus HepG2 or MHCC97H cells treated with control. **B** Western blot analysis of HepG2 and MHCC97H cells treated with different concentrations of CPC for 48 h. β-actin was used as a loading control. The graph shows the relative protein level (lower panel). Student’s t-test was used to compare two groups. *p < 0.05, **p < 0.01 versus HepG2 or MHCC97H cells treated with control.

Flow cytometry (FACS) was used to determine the effect of different doses of CPC on apoptosis in MHCC97H and HepG2 cells. CPC treatment significantly increased the apoptosis of MHCC97H and HepG2 cells (Fig 4A). Furthermore, the apoptotic rate also increased with increasing concentration of CPC (Fig 4A). Therefore, CPC significantly promotes apoptosis of hepatoma carcinoma cells in a dose-dependent manner.

We further demonstrate the mechanism by which CPC enhances HCC cell apoptosis. CPC enhanced the cleavage of full-length PARP1 and promoted the protein levels of pro-apoptotic proteins, including cleaved-caspase-3, Bax and p53, and inhibited the expression of the anti-apoptotic protein Bcl-xL (Fig 4B). Therefore, CPC promotes apoptosis of HCC cells by regulating the protein levels of apoptosis-related genes.

### CPC regulates EMT

**Fig 5:**
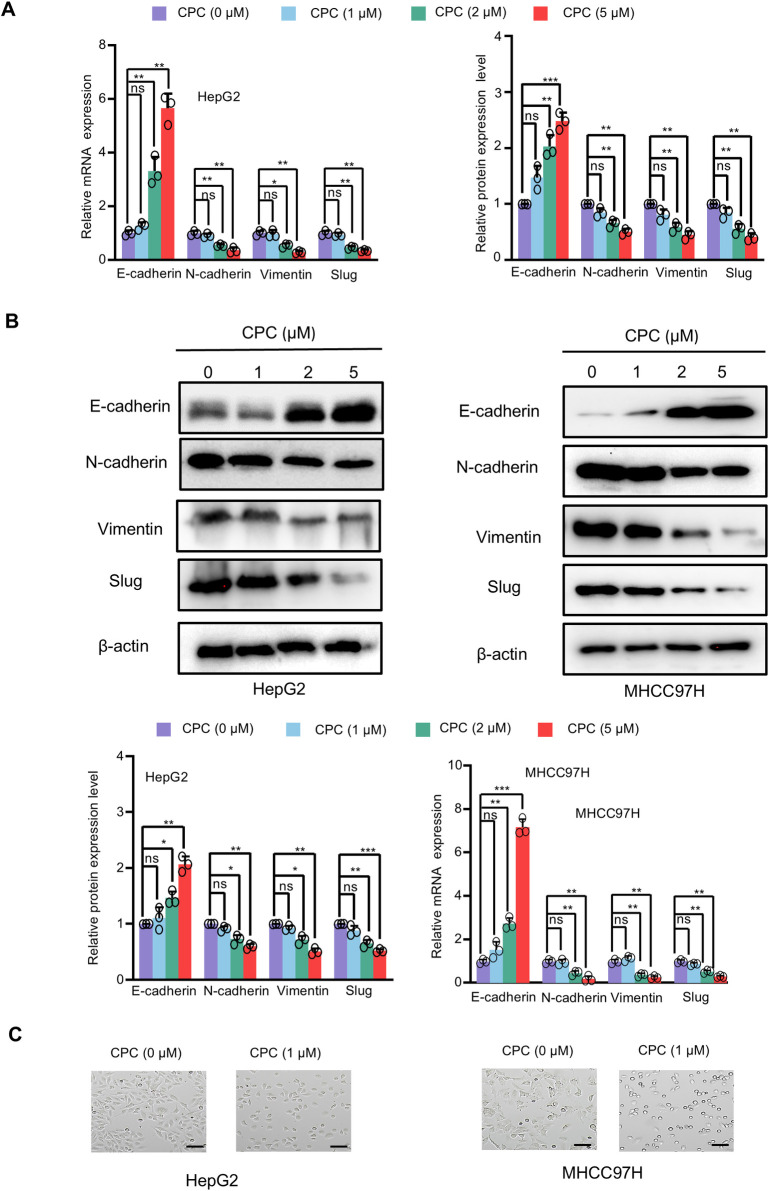
CPC regulates EMT in HCC cells. **A** RT-qPCR analysis of the relative mRNA expression of the indicated genes in HepG2 and MHCC97H cells treated with different concentrations of CPC for 48 h. **B** Western blot analysis of HepG2 and MHCC97H cells treated with different concentrations of CPC for 48 h. The graph shows the relative protein level (lower panel). **C** HepG2 and MHCC97H cells were treated with CPC for 7 days, the morphological changes are shown in the photographs. Scale bar: 100 μm. Data shown are mean ± SD of triplicate measurements that have been repeated three times with similar results. Student’s t-test was used to compare two groups. *p < 0.05, **p < 0.01 versus HepG2 or MHCC97H cells treated with control.

We further determine the mechanism by which CPC inhibits HCC metastasis and invasion. EMT regulators play an important role in cancer cell invasion and metastasis. We found that CPC enhanced the mRNA and protein levels of the EMT marker E-cadherin, and inhibited the mRNA and protein levels of the EMT markers Slug, N-cadherin and Vimentin in a dose-dependent manner (Fig 5A and 5B). Furthermore, CPC treatment inhibited the morphological changes from an epithelial phenotype to a fibroblastoid phenotype (Fig 5C). Therefore, CPC inhibited HCC invasion and metastasis by regulating EMT.

### CPC suppresses breast tumor growth and metastasis *in vivo*

**Fig 6:**
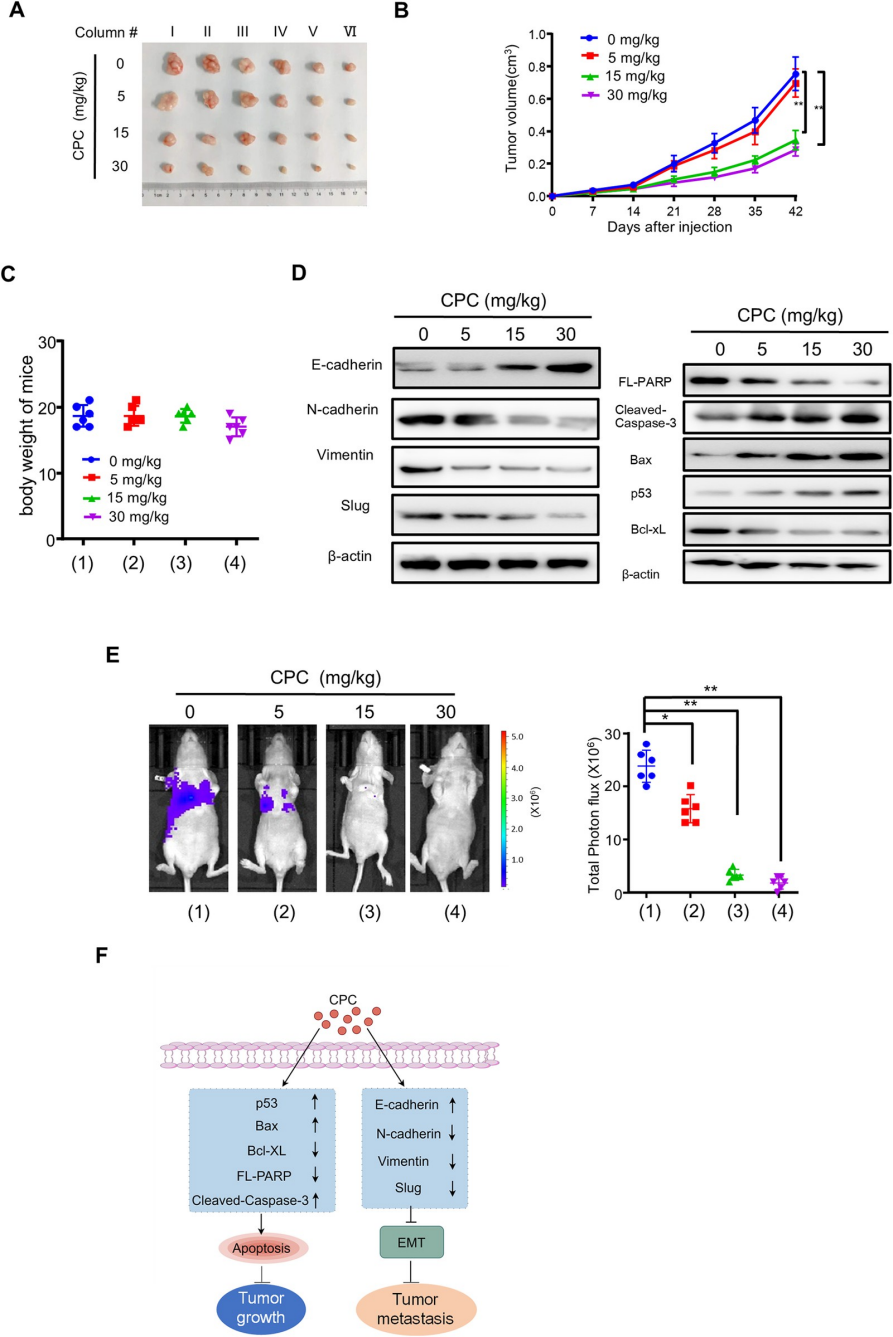
CPC suppresses breast tumor growth and metastasis *in vivo*. **A** Tumor images of HepG2 cells injected subcutaneously into nude mice. **B** The tumor growth curves from (A) measured every 7 days (n = 6). **C** Tumor weights of nude mice from (A). **D** Representative Western blot using tumor tissues from (A) (column III). **E** Representative bioluminescence images (left panel) and bioluminescence analysis (right panel) of the tail vein of nude mice injected with MHCC97H-luc cells treated as in (A). **F** Graphic summary of CPC inhibition of HCC tumor growth and metastasis. CPC induced apoptosis and inhibited EMT by regulating the expression of apoptosis-related genes (p53, Bax, Bcl-XL, etc.) and EMT-related genes (Slug, E-cadherin, N-cadherin, and Vimentin) in a dose-dependent manner, thereby inhibiting tumor growth and metastasis. *p < 0.05, **p < 0.01.

Since CPC inhibits HCC cell proliferation and motility *in vitro*, we further determined the effect of CPC on HCC growth and metastasis *in vivo*. Similar to the results *in vitro*, CPC strongly suppressed HCC tumor growth without affecting the body weight of the mice (Fig 6A–6C). Consistent with the *in vitro* results, CPC regulated EMT-related proteins, and promoted the cleavage of full-length PARP1 and the protein levels of pro-apoptotic proteins, and inhibited the expression of the anti-apoptotic protein Bcl-xL (Fig 6D). Using nude mice metastasis model, we found that CPC significantly inhibited the lung metastasis of HCC cells (Fig 6E). Taken together, these results indicated that CPC inhibits HCC tumor growth and lung metastasis.

## Discussion

Due to the lack of effective chemotherapeutic agents for HCC, it is crucial to screen for effective and safe agents for HCC treatment [32]. In this study, we successfully screened CPC and further investigated that CPC dose-dependently inhibited HCC tumor growth and metastasis *in vitro* and *in vivo*. Mechanistically, CPC promoted apoptosis and inhibited invasion in HCC cells by regulating the expression of apoptosis- and EMT-related genes at the mRNA and protein levels. More importantly, the IC_50_ of CPC in normal cells is much higher than that in cancer cells, predicting a lower side effect of CPC. In conclusion, CPC may be an effective drug for the treatment of hepatocellular carcinoma.

The high mortality rate of HCC patients is mainly due to the presence of metastases in these patients at the time of diagnosis [1, 6, 8]. There are still no effective drugs for the treatment of HCC recurrence and metastasis [8, 9]. We investigated the effect of CPC on the ability of HCC cells to migrate and invade using wound healing and transwell assays *in vitro*. The results showed that CPC inhibited the migration and invasion of HCC cells by regulating EMT, a key step in cancer invasion and metastasis, suggesting that it is a potential novel therapeutic candidate for the treatment of cancer metastasis. Importantly, using the nude mouse metastasis model, we found that CPC inhibited lung metastasis of HCC cancer *in vivo*.

Apoptosis is a form of programmed cell death designed to eliminate unwanted cells during eukaryotic development [33]. Dysregulation of apoptosis is a hallmark of cancer [30, 34, 35]. Therefore, strategies targeting apoptosis are currently promising in cancer therapy and in overcoming chemotherapy resistance in cancer cells. Our results showed that the expression levels of p53, Bax and cleaved caspase-3 proteins were significantly upregulated with increasing concentration of CPC, suggesting that CPC may inhibit the viability of HCC cells and induce apoptosis in HCC cells through activation of the tumor suppressor gene p53.

In hepatocellular carcinoma (HCC), epithelial-mesenchymal transition (EMT) plays a key role in metastasis by enhancing their motility, invasiveness, and resistance to apoptosis [36]. This process involves downregulation of epithelial markers (E-cadherin) and upregulation of mesenchymal markers (N-cadherin, vimentin), allowing cells to migrate and invasive [37]. A variety of signaling pathways, including TGF-β and Wnt/β-catenin and transcription factors (ZEB1, Snail and Twist) mediate EMT of HCC. Targeting the EMT pathway is a therapeutic intervention for HCC metastasis [38, 39]. In this study, we found that CPC significantly inhibited the EMT of HCC cells by decreasing Slug expression, thereby promoting the expression of E-cadherin and inhibiting the expression of N-cadherin and vimentin *in vivo* and *in vitro*, suggesting that CPC is a potential agent for the treatment of HCC metastasis.

In conclusion, our study demonstrated that CPC induced apoptosis and inhibited EMT by regulating the expression of apoptosis-related genes (p53, Bax, Bcl-XL, etc.) and EMT-related genes (Slug, E-cadherin, N-cadherin, and Vimentin) in a dose-dependent manner, thereby inhibiting the proliferation, invasion, and metastasis of HCC cells *in vivo* and *in vitro*, suggesting that CPC is a potential agent for HCC (Fig 6F).

## Supporting information

S1 File Raw data of the cell migration. (XLSX)

S2 File Original western blot. (PDF)
