# Supplementary figures and images for: Cetylpyridinium chloride inhibits hepatocellular carcinoma growth and metastasis through regulating epithelial-mesenchymal transition and apoptosis
Source: PLoS One. 2024 Sep 20;19(9):e0310391. doi: 10.1371/journal.pone.0310391 (PMC11414990; doi:10.1371/journal.pone.0310391)

Fig. 4B

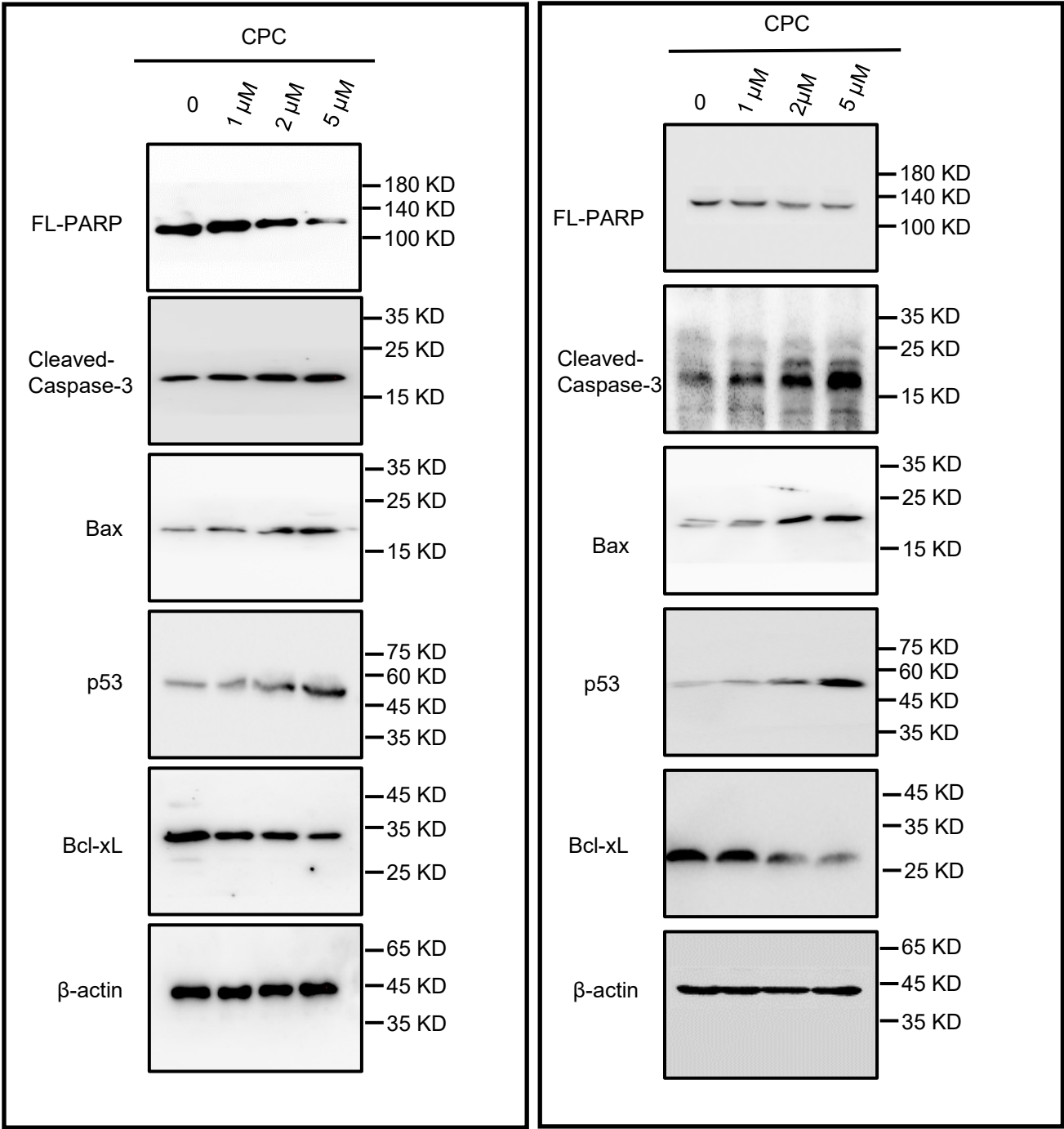

Fig.5B

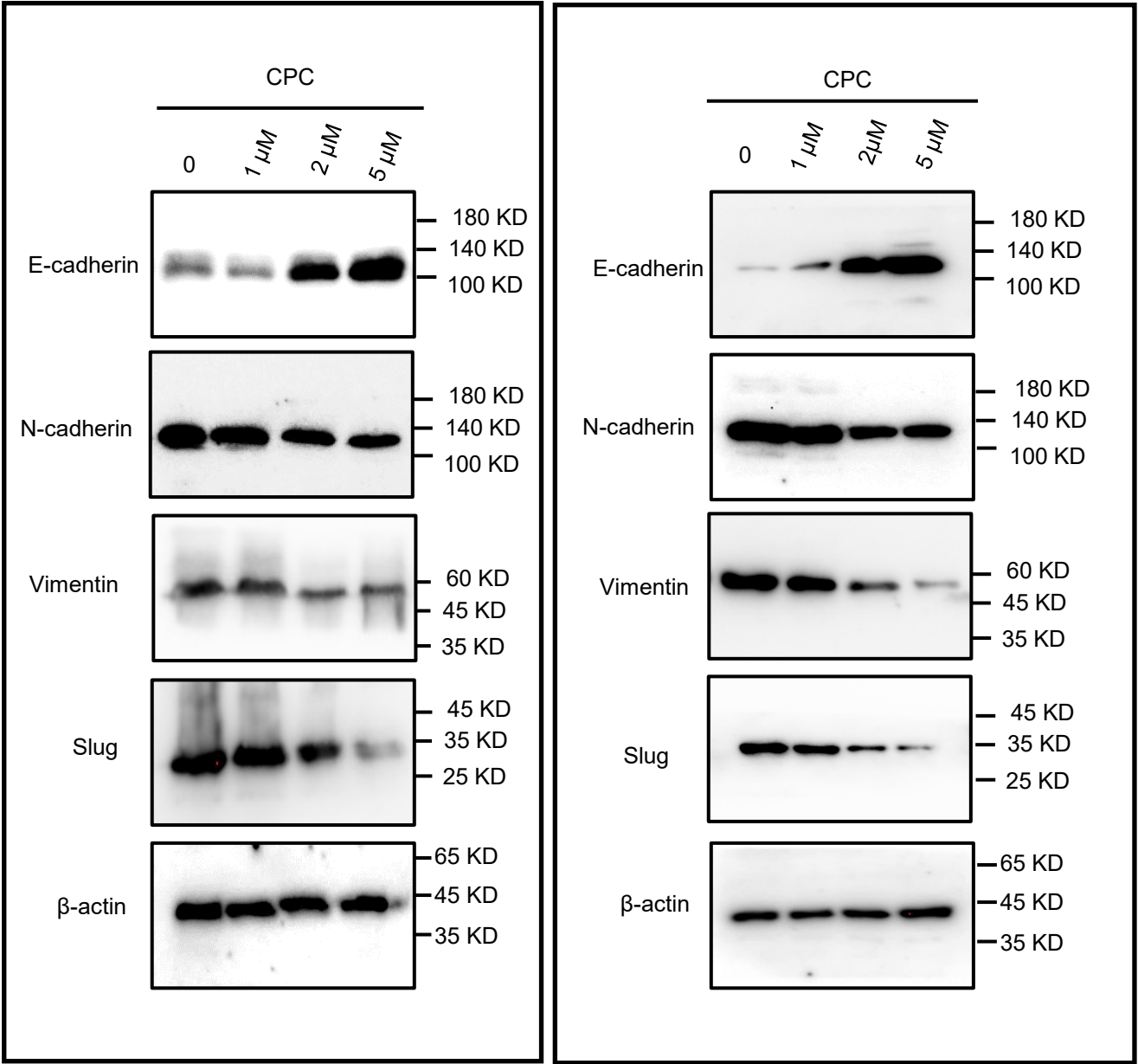

HepG2

MHCC97H

Fig.6D

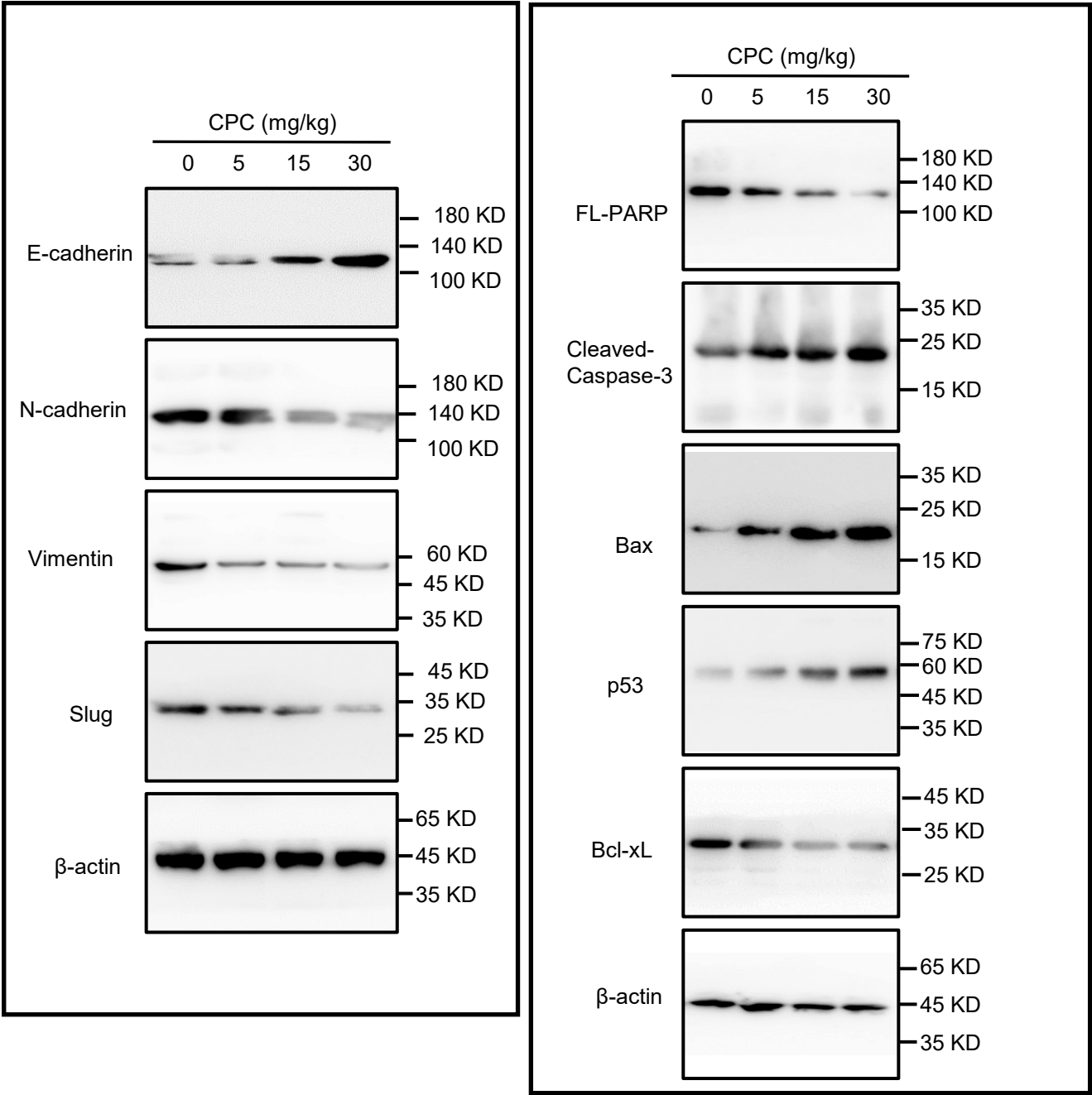

Supplement: S2 File — (PDF) [file pone.0310391.s002.pdf]
